# Supplementary material for: Modeling glioblastoma heterogeneity as a dynamic network of cell states
Source: Mol Syst Biol. 2021 Sep 16;17(9):e10105. doi: 10.15252/msb.202010105 (PMC8444284; doi:10.15252/msb.202010105)
Supplement: Supplementary file 6 — Source Data for Figure 5 [file MSB-17-e10105-s004.zip › Figure5A_sourcedata/GSEA_3017/hallmarks_stateD.GseaPreranked.1621934542601/HALLMARK_GLYCOLYSIS.html]

Details for gene set HALLMARK\_GLYCOLYSIS[GSEA]

|  || Dataset | state23017 |
| Phenotype | NoPhenotypeAvailable |
| Upregulated in class | na\_neg |
| GeneSet | HALLMARK\_GLYCOLYSIS |
| Enrichment Score (ES) | -0.413752 |
| Normalized Enrichment Score (NES) | -1.7087305 |
| Nominal p-value | 0.0259887 |
| FDR q-value | 0.09261035 |
| FWER p-Value | 0.414 |
Table: GSEA Results Summary

  

Fig 1: Enrichment plot: HALLMARK\_GLYCOLYSIS      
 Profile of the Running ES Score & Positions of GeneSet Members on the Rank Ordered List

  

| PROBE | GENE SYMBOL | GENE\_TITLE | RANK IN GENE LIST | RANK METRIC SCORE | RUNNING ES | CORE ENRICHMENT || 1 | MIF |  |  | 48 | 0.351 | -0.0076 | No |
| 2 | STC1 |  |  | 67 | 0.322 | 0.0055 | No |
| 3 | LDHA |  |  | 92 | 0.297 | 0.0119 | No |
| 4 | GPC1 |  |  | 213 | -0.255 | -0.0594 | No |
| 5 | SDC1 |  |  | 300 | -0.264 | -0.1037 | No |
| 6 | HMMR |  |  | 319 | -0.266 | -0.0953 | No |
| 7 | B3GAT1 |  |  | 379 | -0.271 | -0.1181 | No |
| 8 | HS2ST1 |  |  | 477 | -0.282 | -0.1694 | No |
| 9 | ALDH9A1 |  |  | 483 | -0.283 | -0.1495 | No |
| 10 | SDC3 |  |  | 744 | -0.329 | -0.3226 | No |
| 11 | CHPF |  |  | 863 | -0.360 | -0.3836 | Yes |
| 12 | GPC4 |  |  | 891 | -0.369 | -0.3734 | Yes |
| 13 | SAP30 |  |  | 905 | -0.376 | -0.3519 | Yes |
| 14 | RRAGD |  |  | 928 | -0.386 | -0.3366 | Yes |
| 15 | VLDLR |  |  | 1004 | -0.416 | -0.3596 | Yes |
| 16 | CYB5A |  |  | 1014 | -0.422 | -0.3311 | Yes |
| 17 | PAM |  |  | 1055 | -0.448 | -0.3244 | Yes |
| 18 | DEPDC1 |  |  | 1074 | -0.457 | -0.3000 | Yes |
| 19 | CHST2 |  |  | 1081 | -0.460 | -0.2660 | Yes |
| 20 | FUT8 |  |  | 1108 | -0.472 | -0.2464 | Yes |
| 21 | SDC2 |  |  | 1200 | -0.580 | -0.2680 | Yes |
| 22 | PLOD2 |  |  | 1215 | -0.615 | -0.2272 | Yes |
| 23 | CENPA |  |  | 1243 | -0.679 | -0.1911 | Yes |
| 24 | CD44 |  |  | 1270 | -0.762 | -0.1472 | Yes |
| 25 | AURKA |  |  | 1271 | -0.763 | -0.0832 | Yes |
| 26 | CDK1 |  |  | 1311 | -1.433 | 0.0070 | Yes |
Table: GSEA details [plain text format]

  

Fig 2: HALLMARK\_GLYCOLYSIS: Random ES distribution      
 Gene set null distribution of ES for **HALLMARK\_GLYCOLYSIS**
